# Utilizing an In Vitro Fermentation Model to Assess Probiotics on *Eimeria*-Disturbed Cecal Microbiome and Metabolome

**DOI:** 10.3390/ani16020245

**Published:** 2026-01-14

**Authors:** Yani Wu, Xueting You, Shuping Huang, Ju Chai, Yongqi Zeng, Haitao Shi, Xi Wang

**Affiliations:** 1 Key Laboratory of Qinghai Tibetan Plateau Animal Genetic Resource Reservation and Utilization, Southwest Minzu University, Chengdu 610041, Chinahuangshuping1013@163.com (S.H.); shihaitao@swun.edu.cn (H.S.); 2 Key Laboratory of Animal Science of National Ethnic Affairs Commission of China, Southwest Minzu University, Chengdu 610041, China; 3 Department of Animal Sciences, Wageningen University & Research, 6708 PB Wageningen, The Netherlands; ting.you@wur.nl; 4 College of Animal Science and Technology, China Agricultural University, Beijing 100083, China

**Keywords:** probiotics, *Eimeria*, in vitro, microbiota, microbial metabolites

## Abstract

Rectifying the microbiome perturbed by *Eimeria* invasion might alleviate the adverse effects of coccidia on broiler growth. This study employed an in vitro fermentation model to investigate the direct, host-independent effects of two probiotics—*Lactobacillus rhamnosus* (LR) and *Bacillus subtilis* (BS)—on the cecal microbiome and metabolome perturbed by *Eimeria tenella*. Four in vitro fermentation treatments consisted of a healthy control (cecal slurry samples from health broilers), an *Eimeria*-disturbed control (slurry samples from infected broilers), an LR treatment (*Eimeria*-infected slurry + 3 × 10^5^ of LR cfu/mL), and a BS treatment (*Eimeria*-disturbed group + 3 × 10^5^ of BS cfu/mL). 16S rRNA sequencing and metabolomic analysis revealed that *Eimeria* infection resulted in an increase in microbial alpha diversity, promoted opportunistic pathogens, including *Helicobacter* and *Bacteroides*, and suppressed commensals like *Lactobacillus*, concurrently altering 530 intracellular metabolites. Probiotic supplementation partially restored microbial composition. Notably, LR inoculation rectified 107 metabolites across pathways including galactose metabolism and phosphotransferase systems, primarily affecting membrane phospholipid balance. In contrast, BS addition restored only 64 metabolites, largely related to secondary metabolism. The current in vitro study indicates that LR can directly modulate key metabolic disturbances in a dysbiotic microbiota, while the BS may be more dependent on host-mediated interactions.

## Simple Summary

This study used an in vitro model to test how two probiotics affect a chicken gut microbiome interrupted by *Eimeria*. We found that the coccidial infection increased harmful bacteria in the cecal microbiota and disrupted bacterial metabolism. Adding *Lactobacillus rhamnosus* directly helped restore bacterial balance and corrected disrupted metabolites linked to cell membranes. *Bacillus subtilis* also improved bacterial balance but was less effective at fixing the metabolic problems. The results suggest that LR can directly repair partial microbial damage, while BS’s full benefits may depend on the host’s response.

## 1. Introduction

Avian coccidiosis, an intestinal disease caused by the protozoan *Eimeria*, leads to substantial economic losses in poultry production worldwide [1,2]. Following the oral ingestion of *Eimeria* oocysts, infective sporozoites are released within the intestinal tract, invade enterocytes, and disrupt absorption. This damage results in intestinal nutrient leakage and promotes the proliferation of harmful bacteria [3]. The previous omics investigations have indicated that the microbiome in *Eimeria*-infected broilers shifts toward a pathogenic enterotype by enriching abundances of *Lanchnoclostridium*, *Bacillaceae*, *Escherichia*-*Shigella*, and *Proteobacteria* [4,5]. Typically, the intestinal barrier damaged by *Eimeria* is susceptible to secondary infection by *Clostridium perfringens* [6,7], which exacerbates inflammation and can precipitate the onset of necrotic enteritis. These pathological changes collectively impair growth rate and feed efficiency in broilers [8,9]. Therefore, rectifying the perturbed microbiome could potentially mitigate the adverse consequences of *Eimeria* infection on the performance of broilers [5].

Probiotics, as mediators of the intestinal microbiome, have the potential to alleviate the intestinal microbiota disrupted by *Eimeria* infection [10,11]. However, the intestinal microbiota responses to probiotic cells were investigated mainly relying on live chicken trials [3,5,12]. In such in vivo settings, it is difficult to focus solely on the direct microbial response to probiotic cells due to the confounding effects of host-related factors [13,14]. As an alternative, both continuous [13] and static [15] in vitro fermentation systems have been proposed as fast, reproducible tools for directly assessing broiler microbiota metabolism and composition under controlled, pseudo-steady-state conditions [16]. In the current study, we aimed to apply an in vitro culturing approach to closely examine how *Eimeria*-disturbed cecal microbiota, responds to two functionally distinct probiotics, acid-producing *Lactobacillus rhamnosus* (LR) and antimicrobial peptide-producing *Bacillus subtilis* (BS).

The dietary inclusion of LR has been shown to mitigate *Escherichia-Shigella* and *Enterococcus* in broilers through the production of lactic acid [17]. A previous study indicated that LR probiotic alleviated intestinal injury by enhancing the expression levels of the bacterial genes *But* and *Buk*, thereby promoting butyrate production [18]. Similarly, BS, which produces the antimicrobial peptide bacitracin, is a well-documented probiotic known to inhibit intestinal pathogens like *Clostridium perfringens* [19] and *Salmonella Typhimurium* [20] while promoting the growth of beneficial genera such as *Bifidobacterium* [21] and *Ruminococcus* [22]. Given these distinct modes of action, we hypothesized that directly supplementing the *Eimeria*-induced imbalanced microbiota with LR or BS would alleviate the disturbance through distinct mechanisms.

## 2. Materials and Methods

### 2.1. Probiotic Strains

The LR probiotic cells (ATCC7469, Shanghai Luwei Technology, Shanghai, China) were revived in the de Man, Rogosa, and Sharpe broth (MRS, Haibo Biological, Qingdao, China) in an anaerobic fermentation at 37 °C. The BS probiotic cells (CMCC63501, Haibo Biological, Qingdao, China) were revived by nutrient broth (Remel, Lenexa, KS, USA). Pure LR and BS cell suspensions were obtained after three times of PBS washing and centrifugation (450× *g*) and enumerated onto MRS or commercial Bacillus agar plates (Haibo Biological), respectively. Afterward, the cells were resuspended in PBS and diluted to 3 × 10^7^ cfu/mL at stocking density.

### 2.2. Cecal Culture Preparation

This study was conducted at an experimental farm in the College of Animal and Veterinary Sciences at Southwest Minzu University. All experimental protocols and animal handling procedures were approved by the Institutional Animal Ethics Committee at Southwest Minzu University (SMU-202409007, 1 September 2024). A total of 50 one-day yellow broilers were randomly assigned into five floor pens with 10 chicks per pen. Yellow broilers, known as yellow-feathered chicken, refer to Chinese local poultry breeds with higher yellow pigmentation in feather, bone, skin, and enriched aroma profiles [23,24]. Each floor pen was equipped with one nipple drinker, a feeder, and wood shaving litter. A rubber divider was used to avoid potential cross-contamination. The chicks had free access to water and commercial crumble feed. The crumble feed was formulated as corn and soybean meal basal diet without antibiotic inclusion. Nutrient density meets the nutrient requirements of yellow broilers [25]. On day 10, each of the 40 chicks was crop-gavaged with 0.5 mL of PBS containing 5 × 10^4^ *Eimeria tenella* oocysts (a field strain from Sichuan, China), while another 10 chicks received an aliquot of PBS.

According to our previous study, the highest oocyst output was observed on day 8 post-infection of this field strain of *E. tenella* [5]. Typical petechiae at the section of cecum (Merck veterinary manual, Rahway, NJ, USA) was also confirmed to ensure the coccidial infection at 8 d post-challenge. Cecal slurry samples from two chicks were aseptically collected and pooled into a sterile WirlPak bag. Each slurry sample was weighed and ten-fold diluted with anaerobic PBS in an anaerobic workstation (10% CO_2_, 5% H_2_, and 85% N_2_, Electrotek, West Yorkshire, UK). The slurry mixture of two chicks served as one biological replicate since the volume of cecal content from one bird at 18 days of age was not enough for the bellowing in vitro system.

### 2.3. In Vitro Fermentation and Treatment

In the preliminary study, multiple media were compared to assure stability of microbial composition and metabolites in an immobilized fermentation system. The modified Viande Levure (VL) medium helped 250 out of 298 gena in cecal microbiota, reviving in 24 h of incubation. The standard VL medium was widely applied in previous in vitro fermentation of chicken microbiota [13,26]. In the present study, porcine mucin, fructooligosaccharides, vitamin, and uric acid were added to the standard recipe to assure the growth and metabolism need of chicken cecal microbiota [13]. One liter of modified VL medium contained 10 g of tryptose, 5 g of yeast extract, 5 g of NaCl, 4.5 g of KCl, 2.5 g of glucose, 2.5 g of fructo-oligosaccharides, 2.4 g of beef extract, 2 g of mucin, 1.5 of NaHCO_3_, 1 mL of Tween 80, 1 mL of vitamin solution, 0.8 g of L-cysteine hydrochloride, 0.7 g of uric acid, 0.6 g of MgSO_4_, 0.5 g of KH_2_PO_4_, 0.4 g of bile salt, 0.2 g of MnCl_2_·4H_2_O, 0.05 mL of hemin solution, 0.005 g of FeSO_4_·7H_2_O, 959.3 mL of distilled water, and pH 7.0. All components were purchased from Solarbio (Beijing, china), except bile salts (Sigma-Aldrich, Shanghai, China), yeast extract (Sigma-Aldrich), NaHCO_3_ (Fischer Scientific, Pittsburgh, PA, USA), fructo-oligosaccharide, and tween 80 (Biosharp life sciences, Hefei, China).

Four in vitro fermentation treatments consisted of a healthy control (cecal slurry samples from health broilers), an *Eimeria*-disturbed control (slurry samples from infected broilers), an LR treatment (*Eimeria*-infected slurry + 3 × 10^5^ of LR cfu/mL), and a BS treatment (*Eimeria*-disturbed group + 3 × 10^5^ of BS cfu/mL). The working concentration of probiotics applied in this in vitro model was determined based on the recovery concentration of BS or LR measured in the cecal content of broilers from our previous in vivo trial [5]. According to the above treatment setting, each of the 30 mL fermentation systems (3 mL of cecal slurry + 27 mL of modified VL medium) were inoculated with 0.3 mL of PBS, LR or BS stocking solution. The 3 mL of slurry mixture was collected from two individual birds (served as one biological replicate). The in vitro intestinal simulator (Jiade Precision Techonology, Beijing, China) consisted of four independent glass vessels which allowed us to run four treatments per batch. Each fermentation treatment was undergone in one of the four vessels, and in vitro fermentation was run for five batches (batch served as block factor) at a microaerobic atmosphere (5% O_2_, 10% CO_2_, 85% N_2_) at 42 °C, 150 RPM for 24 h [15]. In the preliminary study, the fermentation times of 0, 12, 24, 36, and 48 h were evaluated for bacteria enumeration and diversity (16S rRNA sequencing). We applied 24 h of in vitro fermentation in the current trial due to the 24 h fermentation achieving similar colonizer numbers and diversity as compared to the original cecal microbiota.

### 2.4. Microbial and Metabolome Sampling

During the fermentation (0, 12, 24 h), microaerobic counts were enumerated onto LB agar plates with 5% O_2_, 10% CO_2_, and 85% N_2_. At the end of the fermentation, a pellet of 10 mL of fermentation aliquot was obtained at a centrifugation of 5000× *g* for 10 min, dip-frozen in liquid nitrogen, and stored at −20 °C for 16S rRNA sequencing. The intracellular metabolites were collected via differential centrifuge method [27]. Impurities and culture residues were removed by 1000× *g* for 10 min. The supernatant was transferred to another sterile falcon tube and subjected to a second centrifugation at 5000× *g* for another 10 min. The bacterial pellet was collected, dip-frozen in liquid nitrogen, and stored at −80 °C for subsequent intracellular metabolomics analysis.

### 2.5. 16S rRNA Sequencing and Bioinformatics

Microbial diversity and composition were evaluated by 16S rRNA sequencing [25]. Briefly, genomic DNA was collected using a commercial kit (QIAamp Fast DNA Stool Mini Kit, Qiagen, Germantown, MD, USA). The quantity and quality of genomic DNA were measured by the Nanodrop 1000 spectrophotometer (Wilmington, DE, USA) and 0.8% agarose electrophoresis gel. The V3–V4 region (~550 bp) of microbial 16S rRNA was amplified by 338F forward primer (5′ACTCCTACGGGAGGCAGCAAG) and 806R reverse primer (5′GGACTACHVGGGTWTCTAAT). The purity of target amplicons was confirmed on 1% agarose electrophoresis gel and recollected by QIAquick Gel Extraction Kit (Qiagen). A pair of indexes was labeled to the overhang sequence of amplicons from identifiable birds; thus, bacterial sequences could be tracked by bird. The final PCR results were quantified by the Bioanalyzer DNA 1000 chip (Agilent Technologies, Inc., Santa Clara, CA, USA) and read by the Illumina HiSeq system in a commercial lab (MajorBio, Shanghai, China).

Raw sequencing reads underwent quality control by FastQC (0.12.1). The low-quality reads were filtered out according to a previous study [28]. Briefly, raw reads shorter than 110 nt, mismatches to described barcodes, one end of primers, and reads harboring more than 7% of low-quality bases (Phred quality threshold < 20) were trimmed and removed. The following sequence analysis was analyzed using the QIIME 2 v1.9.0.: denoising approach by DADA2, filtering of contigs by length (at least 420 bp), chimera deletion (USEARCH v6.1), and dereplication. Qualitied clean reads were achieved by removing the adapter sequence of FASTA data (Trimmomatic), and clean copies were classified into individual birds via PEAR software v0.9.6. Sequences with ≥97% similarity were assigned to typical operational taxonomic units (OTU). To limit data size effect, similar reads (around 42,000 reads) per sample were randomly selected for further analysis. Sequences were BLAST against the Silva database for bacterial taxonomy and are available at https://www.ncbi.nlm.nih.gov/sra/PRJNA1394992 (available on 7 January 2026). Alpha diversity indexes at the genus level (Sobs, Shannon, and Chao1) were collected via Mothur software (version 1.30.2), and Bray–Curtis distance matrices were calculated via Qiime software (version 1.9.1), followed by principal coordinate analysis (PCoA). Linear discriminant analysis (LDA) was used for dimensionality reduction, and an LDA score larger than 3.5 was used for LEfSe analysis to differentiate bacteria composition.

### 2.6. Untargeted Metabolomics and Bioinformatics

Intracellular metabolites from bacterial pellets were analyzed via liquid chromatography–mass spectrometry method in a commercial lab (Allwegene Tech., Nanjing, China). Raw data were converted to mzML format using ProteoWizard software (v 3.0.7414) and then processed using an in-house developed R package (with XCMS as the core, v4.6.0) for peak detection, peak extraction, peak alignment, and integration. Subsequently, metabolite annotation was performed using an internal MS2 database (Allwegene). Matching was carried out for compound annotation, and the algorithm score cutoff value was set to 0.70.

To distinguish the impacts of *Eimeria* invasion in cecal bacterial metabolites, a volcano plot was created to distinguish metabolites with VIP > 1.0 and *p* < 0.05 between healthy controls and Eimeria infection controls. Differential metabolites with a log2 fold change larger than 1.5 were selected and donated with bacterial KEGG pathway. Probiotic impacts on metabolites of the *Eimeria*-disrupted microbiome were also evaluated by comparing metabolome in *Eimeria* birds with that of *Eimeria*-BS or *Eimeria*-LR groups.

### 2.7. Data Analysis

A randomized complete block design was applied in this study to evaluate the directive addition of probiotics to in vitro cecal microbiota, and the batch of fermentation served as a block factor (replication). The alfa diversity indexes and dominant bacterial compositions (relative abundance) were analyzed using the GLM procedure in SAS 9.4 (SAS Institute, Inc., Cary, NC, USA). The following statistical model was used: *Y_ij_* = *μ* + *α_i_* + *β_j_* + *ε_ij_*_,_ where *Y_ij_* represents above dependent variables, *μ* denotes population mean, *α_i_* denotes probiotic treatment (fixed factor), *β_j_* denotes batch (random factor), and *ε_ij_* denotes random error. Spearman correlations were conducted to reveal relationships between differentiated microbials and intracellular metabolites. The significance level was set as *α* = 0.050.

## 3. Results

### 3.1. Microbial Diversity

**Figure 1:**
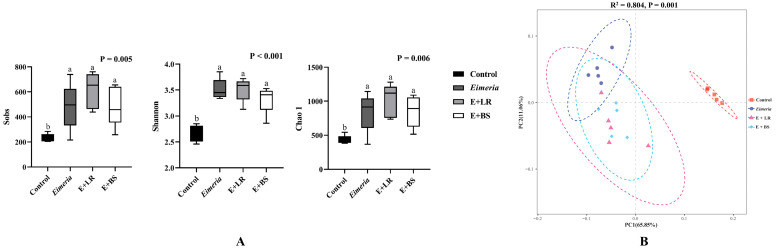
Effects of probiotics on alpha diversity (**A**) and beta diversity (**B**) in cecal microbiota at genus level via an in vitro fermentation platform (*n* = 5). Alpha diversity indexes were analyzed in ANOVA analysis with α = 0.050 (*n* = 5). Beta diversity was analyzed using the principal coordinate analysis based on Bray–Curtis dissimilarity metrics and confirmed with adonis analysis. a–b: the violin boxes not sharing a common subscript are considered different at α = 0.050.

Alpha diversity indexes and beta diversity (PCoA analysis) are presented in Figure 1. *Eimeria* infection resulted in higher bacterial diversity by increasing Sobs, Shannon, and Chao 1 at the genus level, as compared to healthy control birds (*p* = 0.005, <0.001, and 0.006), and dramatically shifting bacterial composition by clustering in a distinct position from healthy controls (*p* = 0.001). However, adding LR or BS shows a trend of drafting microbial composition towards to health control according to the PCoA analysis, without significantly reducing bacterial diversity or richness.

### 3.2. Microbial Compositions

**Figure 2:**
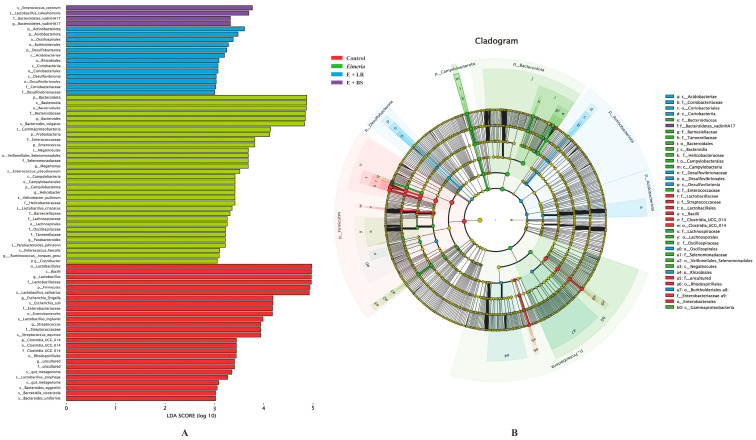
The LefSe analysis (**A**) of cecal microbiota using LDA scores of 3.5 or greater and the cladogram (**B**). Different-colored regions represent different groups. Circles indicate phylogenetic levels from phylum to genus. The diameter of each circle is proportional to the abundance of that group.

The LEfSe (LDA Effect Size) analysis in Figure 2 presents dominated taxa among four groups. Microbiota in healthy controls (red color bars) was dominated by *Firmicutes*, *Lactobacillus salivarius*, *Lactobacillus prophage*, *Rhodospirillales*, *Clostridia* UCG 014, *Streptococcus equinus*, *Escherichia coli*, *Escherichia Shigella*, *Barnesiella viscericola*, and *Bacteroides eggerthii*. Dominated taxa in *Eimeria*-infected broilers (green color bars) were *Proteobacteria*, *Bacteroides vulgatus*, *Gammaproteobacteria*, *Enterococcus*, *Negativicutes*, *Selenomonadaceae*, *Megamonas*, *Enterococcus pseudoavium*, *Campylobacteria*, *Helicobacter pullorum*, *Lactobacillus crispatus*, *Coprobacter*, and *Ruminoccus torques*. Adding LR to this *Eimeria*-disturbed microbiota increased the abundance of *Acidobacteriae*, *Oscilospirales*, *Burkholderiales*, *Desulfovibrionaceae*, *Rhizobiales*, and *Coriobacteriaceae*, whereas adding BS increased that of *Enterococcus cecorum*, *Lactobacillus coleohominis*, and *Bacteroidetes vadin* HA17.

**Figure 3:**
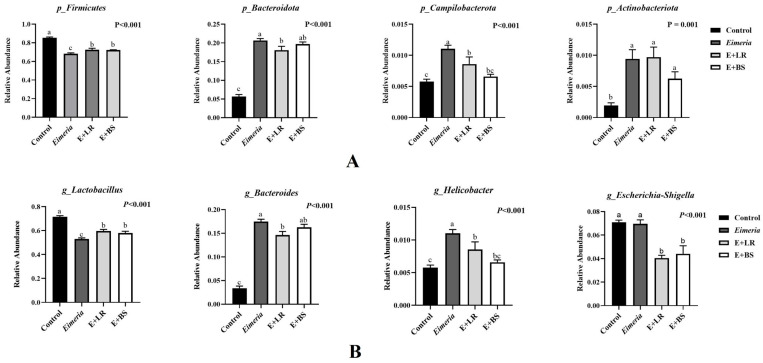
Effects of probiotics on relative abundance levels of dominant microbiota at phylum level (**A**) and genus level ((**B**), *n* = 5). a–c: the violin boxes not sharing a common subscript are considered different at α = 0.050.

The relative abundance (read numbers/total read number) in dominant bacteria are compared at the phylum and genus levels in Figure 3. *Eimeria* infection resulted in more Firmicutes and less *Bacteroidota*, *Campilobacterota*, and *Actinobateriota* in healthy birds (all *p* < 0.050, Figure 3A, *Eimeria* vs. control). Typically, *Lactobacillus*’ relative abundance was reduced in *Eimeria*-infected birds, whereas *Bacteroides* and *Helicobacter* relative abundance levels increased (Figure 3B). Adding LR cells directly to the *Eimeria*-disturbed microbiota partially restored relative levels of *Firmicutes*, *Bacteroidota*, and *Campylobacterota* at phylum level and the proportions of *Lactobacillus*, *Bacteroides*, and *Helicobacter* at the genus level (*Eimeria* vs. *Eimeria* + LR). Adding BS cells to *Eimeria*-disturbed microbiota could also restored the proportion of Firmicutes and *Campylobacterota* at the phylum level and *Lactobacillus* and *Helicobacter* at the genus level (*Eimeria* vs. *Eimeria* + BS).

### 3.3. Differentiated Metabolites

**Figure 4:**
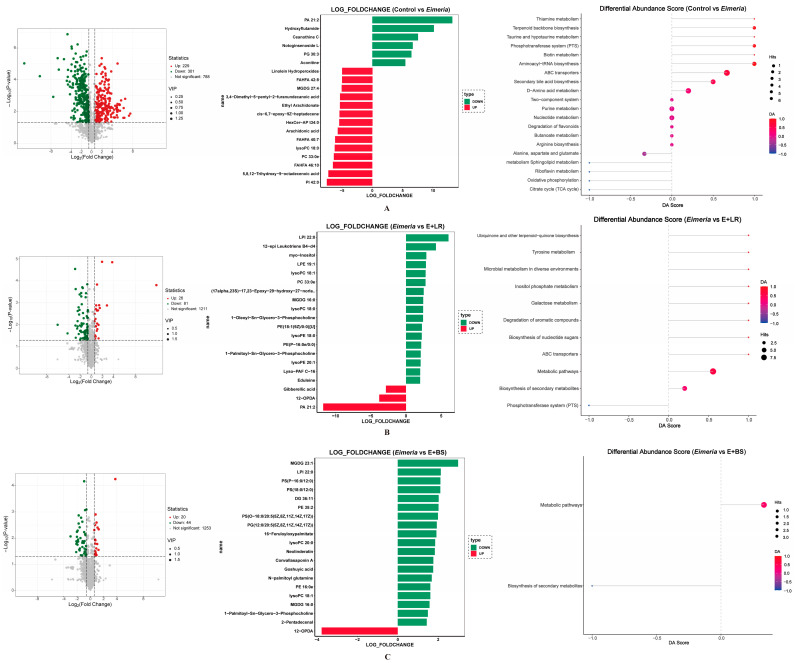
Volcano plots of differential abundant metabolites, fold change in differential metabolites, and KEGG analysis ((**A**): control vs. Eimeria, (**B**): Eimeria vs. E + LR, (**C**): Eimeria vs. E + BS, *n* = 5). Volcano plots illustrate differential metabolites with VIP value > 1.0 and a *p*-value < 0.050. Red dots represent increased metabolites, while green dots represent decreased metabolites in Eimeria-challenged groups (**A**) or the probiotic-supplemented groups (**B**,**C**). The size of the dots corresponds to the VIP value. Log2 fold change in differential metabolites are presented in bar charts. Red bars indicate upregulated metabolites, and green bars indicate downregulated metabolites in *Eimeria*-challenged groups (**A**) or the probiotic-supplemented groups (**B**,**C**). KEGG enrichment analysis results are displayed in a score plot. The *y*-axis shows the enriched KEGG pathways. The *x*-axis represents the differential abundance score. The size of the dots (hits) denotes the number of metabolites enriched in each respective pathway.

Differentiated intracellular metabolites between two of the four in vitro fermentation treatment are presented in Figure 4A (healthy microbiota vs. *Eimeria*-disturbed). *Eimeria*-disturbed microbiota exhibited 530 differential interbacterial metabolites as compared to healthy microbiota. The fold change analysis indicated that *Eimeria* infection decreased phosphatidic acid (PA) 21:2, hydroxyflutamide, ceanothine C, notoginsenoside L, phosphatidylglycerol (PG) 30:3, aconitine, and linolein and increased linolein hydroperoxides, branched fatty acid esters of hydroxy fatty acids (FAHFA) 42:8, monogalactosyldiacylglycerol (MGDG) 27:4, 3,4-dimethyl-5-pentyl-2-furanundecanoic acid, ethyl arachidonate, cis-6,7-epoxy-9z-heptadecene, hexcer-AP t34:0, arachidonic acid, FAHFA 40:7, lyso-phospholipid (lysoPC) 18:0, phosphatidylcholine (PC) 33:0, FAHFA 46:10, 5,8,12-trihydroxy-9-octadecenoic acid, and phosphatidylinositol (PI) 42:0 in cecal microbiota. The microbial KEGG analysis indicated that altered metabolites were involved in thiamine metabolism, terpenoid backbone biosynthesis, taurine and hypotaurine metabolism, phosphotransferase system (PTS), biotin metabolism, aminoacyl-tRNA biosynthesis, ATPase (ABC) transporters, secondary bile acid biosynthesis, D-amino acid metabolism, thiamine, alanine, and aspartate and glutamate metabolisms (Figure 4A).

Interestingly, adding LR probiotics directly into the culture restored 107 metabolites as seen in Figure 4B (*Eimeria* vs. *Eimeria* + LR), and adding BS restored 64 metabolites as seen in Figure 4C (*Eimeria* vs. *Eimeria* + BS). Direct addition of LR probiotic cells to the in vitro *Eimeria*-infected microbiota increased PA 21:2 and decreased lysoPC 18:0 and 18:1, PC 33:0, and MGDG 16:0 (Figure 4B), whereas adding BS probiotic cells to the culture also corrected the levels of MGDG 23:1 and 16:0 and lysoPC 18:0, 18:1, and 20:0. Adding LR increased metabolites enriched in microbial galactose metabolism, inositol phosphate metabolism, ABC transporters, degradation of aromatic compounds, and PTS. The BS-altered metabolites enriched in only two pathways: biosynthesis of secondary metabolites and an undefined metabolic pathway.

### 3.4. Correlation Network Between Microbes and Metabolites

**Figure 5:**
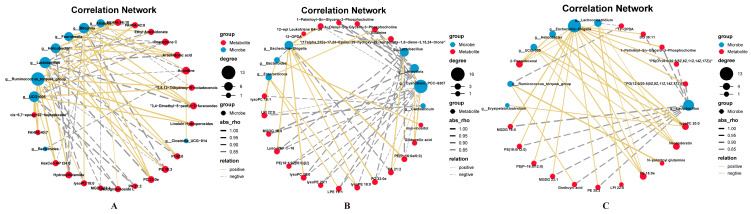
Correlation network analysis between differential microbiota at genus levels and differential metabolites ((**A**): control vs. *Eimeria*, (**B**): *Eimeria* vs. E + LR, (**C**): *Eimeria* vs. E + BS, *n* = 5). Red dots represent differential metabolites, and blue dots represent differential microbes. The size of dots corresponds to the relative degree of abundance of metabolites or microbes. Solid lines indicate positive correlations between microbes and metabolites, whereas the broken line indicates negative correlations. The size of line corresponds to absolute Spearman’s rank correlation coefficient.

The relative abundance levels of differential microbes are strongly corrected to differentiated intracellular metabolites (Figure 5). In *Eimeria*-infected birds, *UCG*-*005* and *Ruminococcus torgues* were negatively correlated to PA 21:2 (r = −0.90 and −0.95, respectively, and *p* < 0.050, Figure 5A), whereas the predominant *Helicobacter* was positively correlated to PI 42:0 and PC 33:0 (r = 0.90 and 0.95, respectively, and *p* < 0.050). Increased *Lactobacillus* in LR-treated microbiota was positively correlated to PA 21:2 and negatively correlated to PC 33:0 (r = 0.95 and −0.90, respectively, *p* < 0.050, Figure 5B), while reduced *Escherichia*-*Shigella* was also associated with decreased PC 33:0 (r = 0.95, *p* < 0.050) in LR-treated microbiota. As compared to the LC probiotics, less correlation relationships were observed in the BS-treated microbiota (Figure 5C).

## 4. Discussion

The in vitro fermentation system employed in this study provided valuable, host-independent insights into the direct interaction between probiotics and the *Eimeria*-disturbed microbiota; it is imperative to acknowledge its inherent limitations. Although the model cannot replicate critical in vivo dynamics such as peristalsis, bile acid secretion, or the precise oxygen gradient of the cecum, all of which shape microbial community structure and function, the modified VL media allowed 250 out of 298 gena in cecal microbiota to revive after 24 h of incubation.

The current study confirmed that *Eimeria tenella* infection disturbed the cecal microbiota, increasing overall microbial diversity (Figure 1) while driving a compositional shift characterized by the expansion of opportunistic pathogens such as *Helicobacter pullorum* and *Bacteroides* (Figure 3). These findings agree with previous studies [3,29,30]. *Helicobacter pullorum*, a pathogen associated with human diarrheal disease, is typically harbored in healthy broiler ceca at low levels [31,32]. However, *Helicobacter* can dominate when birds are under immunodepression or enteritic pathogen invasion [32]. Similarly, *Bacteroides* obligate anaerobes that normally serve as the key degraders of complex carbohydrates and producers of short-chain fatty acids [33]. In addition, derived isovaleric acid has been shown to enhance mucosal immunity [34]. Nevertheless, translocation of *Bacteroides* from the intestinal lumen into the mucosa or bloodstream can induce systemic inflammation due to host responses to their lipopolysaccharide endotoxin [35]. As *Eimeria* infection damages intestinal integrity, leading to nutrient leakage and secondary infection [4,5], this cascade likely drives commensal dysbiosis during coccidiosis [5].

This microbiota dysbiosis is closely linked to profound change in microbial metabolites. Our metabolomic analysis revealed that the *Eimeria*-disturbed microbiota produced 530 differential intrabacterial metabolites, significantly impacting the ABC transporters and PTS pathways (Figure 4). ABC transporters are a diverse superfamily of membrane protein complexes that utilize ATP hydrolysis to power substrate movement across biological membranes or perform mechanical work [36], while the PTS represents a crucial network of proteins that couple phosphoryl transfer to sugar uptake in bacteria. The enrichment of these pathways indicated that the microbiota disrupted by *Eimeria* infection were undergoing substantial energy consumption, enhanced nutrient uptake, and significant biological alterations in intestinal microbes.

In the absence of a host response, probiotic administration exerted minimal influence on overall microbial diversity (alpha diversity) within the *Eimeria*-disturbed microbiota. This contrasts with previous host-dependent effects of probiotics. For instance, oral LR administration has been shown to mitigate heat stress-induced microbial dysbiosis by promoting microbial diversity [37]. Similarly, dietary supplementation with other genera, *Lactobacillus fermentum*, restored ileal diversity and cecal richness which were diminished following *Clostridium perfringens* challenge [38]. The mechanistic basis for such probiotic efficacy is largely mediated through microbial metabolites (e.g., short-chain fatty acids, indole, tryptamine, vitamins, bacteriocins) that facilitate host–microbiota crosstalk, reinforce barrier function, and support immune homeostasis [39]. Correspondingly, both LR and *L. fermentum* attenuated intestinal inflammation by regulating cytokines and T-helper cell responses in birds [37,38]. Additionally, LR promoted intestinal barrier integrity via enhancement of Wnt/β-catenin signaling [37]. These outcomes rely on active host–probiotic interactions, which facilitate probiotic-mediated immunomodulation through the production of natural and antigen-specific antibodies, signaling via toll-like receptors, and regulation of T cells and cytokine networks [39]. Consequently, in the absence of such host interaction, probiotic cells are less effective in modulating cecal microbiota in an in vitro system.

Despite the limited impact on overall diversity, direct LR supplementation partially restored microbial community structure disrupted by *Eimeria* invasion. Specifically, LR recovered population of key commensals, including *Lactobacillus*, *Bacteroides*, and *Helicobacter*, shifting beta diversity (PcoA) toward a community more closely resembling that of healthy birds. It agreed with probiotic administration in *Eimeria*-challenged broiler trials, demonstrating the capacity to promote beneficial taxa and competitively exclude pathogenic species [3]. Beyond competition for nutrients and growth space, *Lactobacillus* strains exert colonization resistance against intestinal pathogens through the production of antimicrobial compounds, including organic acids, hydrogen peroxide, bacteriocins, and biosurfactants [40]. Typically, LR could reduce *Helicobacter* adhesion by downregulating the expression of adhesin gene *SabA* [41] and suppression of the flagellar regulator gene *flgR* and the acid resistance gene *arsS* to reduce *Helicobacter* motility [42].

Through the partial amelioration of microbial dysbiosis, LR probiotics restored a subset of typical microbial metabolites, identifying 107 differential compounds. Specifically, this study revealed an increase in PA and a concomitant decrease in PC and lyso-PC within *Eimeria*-disturbed microbiota. Phosphatic acid (PA) serves as a central precursor for all bacterial membrane phospholipids [43]. In contrast, PC is the predominant membrane-forming phospholipid in eukaryotes, only presenting in 15% of bacteria [44]. Bacterial membranes are primarily composed of phospholipids such as phosphatidylethanolamine, phosphatidylglycerol, and cardiolipin [45]. The observed dysbalanced PA and PC in those *Eimeria*-disturbed microbiota suggests an alteration in the physicochemical properties of bacterial membranes, including fluidity, permeability, and the function of membrane-associated proteins. LR probiotics contributed to the recovery of PA and PC levels, an effect associated with shifts in the relative abundance of *Escherichia*-*Shigella* and *Lactobacillus* (Figure 5B). Notably, although *Escherichia*-*Shigella* typically lacks PC [44], our data revealed a positive correlation between PC levels and *Escherichia*-*Shigella* and a negative correlation with *Lactobacillus*. Thus, these findings indicate that LR probiotics may directly modulate membrane phospholipid homeostasis within the dysbiosis community.

In vitro, BS probiotics acted primarily as a strong competitor, selectively reducing opportunists like *Bacteroides* and *Escherichia-Shigella* while promoting beneficial *Lactobacillus*. This aligns with in vivo broiler trials showing BS suppression of *Bacteroides* and *Escherichia*-*Shigella* [46,47] and enrichment of *Lactobacillus* and *Bifidobacterium* [48]. *Escherichia*-*Shigella* and *Bacteroides* can compromise intestinal integrity and disseminate virulence factors, thereby increasing host susceptibility to infection and diarrhea [4]. The competitive activity of BS is attributed to its capacity to synthesize a broad spectrum of antimicrobial compounds, including bacteriocins, lytic enzymes, polyketides, lipopeptides, siderophores, and volatile organic compounds [49]. This antimicrobial arsenal enables BS to selectively target specific pathogens and opportunistic bacteria. Furthermore, BS supplementation increases concentrations of lactic, succinic, and butyric acids in broiler ileum and ceca [50]. Thus, the decrease in environmental pH likely facilitates the recovery of *Lactobacillus* as a dominant taxon.

In contrast, BS was less effective than LR regarding the modulation of the microbial metabolome, influencing only 64 metabolites and two unclear biological pathways. One possible speculation is that the BS probiotic highly relies on host interaction. In an in vivo condition, BS has been shown at the cellular level to enhance enterocyte proliferation, upregulate MUC2 expression, and reduce proinflammatory responses in birds [51]. BS can produce bioactive metabolites including hypoxanthine, niacin, and pantothenate, which directly interact with host cells [51]. Additionally, BS has also been reported to cause epithelial hypoxia and promote the expansion of short-chain fatty acid producers [48]. Those effects that support intestinal barrier integrity and microbial homeostasis were absent in the current model. The inability of BS to fully correct cecal dysbiosis in this system underscores the constrained probiotic functionality in the absence of cooperative host–microbiome crosstalk, highlighting the critical role of this interaction in driving microbial resilience and metabolic recovery.

## 5. Conclusions

In summary, this study demonstrates that *Eimeria* infection induces significant cecal dysbiosis, characterized by an overgrowth of opportunists and profound alterations in the microbial metabolome, particularly affecting bacterial membrane phospholipid metabolism. While the probiotic *Lactobacillus rhamnosus* directly modulated the disturbed microbiota, partially restored beneficial commensals, and rectified key metabolic imbalances, *Bacillus subtilis* exhibited a more limited ability to influence microbial metabolites under these conditions. The findings underscore that the efficacy of probiotics is contingent upon their specific mechanisms of action. *Lactobacillus rhamnosus* directly influenced microbial community structure and function, whereas *Bacillus subtilis*’ full benefits likely depend on active host–microbe interactions.

## Data Availability

The 16S rRNA sequencing data is available from 7 January 2026 from website: https://www.ncbi.nlm.nih.gov/sra/PRJNA1394992. The metabolomic data sharing is available by contacting the corresponding author by wangxi@swun.edu.cn.

## Abbreviations

The following abbreviations are used in this manuscript:

LR	*Lactobacillus rhamnosus*
BS	*Bacillus subtilis*
VL	Viande Levure
OTU	Operational taxonomic unit
PCoA	Principal coordinate analysis
LDA	Linear discriminant analysis
